# Integrated multi-omics identifies CRP as a prognostic biomarker and reveals complement consumption in HIV-associated AECOPD

**DOI:** 10.3389/fimmu.2026.1855646

**Published:** 2026-06-19

**Authors:** Tao Qin, Wenlong Huang, Changwei Yang, Tianming Lu, Jun Wang, Jie Chen, Linli Chen, Tianyu Yan, Tingting Qian, Hao Yang, Linke Lu, Defa Huang, Minghong Zhao

**Affiliations:** 1 Laboratory Medicine, Guizhou Aerospace Hospital, Zunyi, China; 2 Department of General Medicine, The First People’s Hospital of Zunyi (Third Affiliated Hospital of Zunyi Medical University), Zunyi, China; 3 Department of Nuclear Medicine, The Affiliated Hospital of Zunyi Medical University, Zunyi, China; 4 Guangxi Zhuang Autonomous Region Center for Disease Control and Prevention, Nanning, China; 5 Department of Laboratory Medicine, The Affiliated Yongchuan Hospital of Chongqing Medical University, Chongqing, China; 6 Laboratory Medicine, First Affiliated Hospital of Gannan Medical University, Ganzhou, China

**Keywords:** AECOPD, biomarker, CRP, HIV, immune dysregulation, prognosis

## Abstract

**Objective:**

To delineate the distinct immunometabolic perturbations in HIV-associated acute exacerbation of chronic obstructive pulmonary disease (HIV-AECOPD) and to identify circulating biomarkers predictive of short-term prognosis.

**Methods:**

An integrated proteomic and metabolomic discovery analysis was conducted on serum from patients with HIV-AECOPD, AECOPD alone, and healthy controls (n=5 per group). Key differentially expressed molecules were subsequently validated via targeted assays in a larger, independent cohort (n=20 per group). The prognostic value of validated biomarkers for 3-month poor outcomes was evaluated using a Random Forest model and receiver operating characteristic (ROC) curve analysis.

**Results:**

Multi-omics discovery revealed a unique serum profile in HIV-AECOPD, characterized by dysregulated humoral immunity, complement activation, neutrophil extracellular trap formation, and metabolic reprogramming. Validation assays were performed for six candidate biomarkers (CRP, SAA, IgG, TG, C3, and C4). Among these, CRP emerged as the most important predictor of poor 3-month prognosis (Random Forest Mean Decrease Gini = 1.33), demonstrating significant predictive value (AUC = 0.8125, 95% CI: 0.628–0.997).

**Conclusions:**

This study defines a specific immunometabolic signature in HIV-AECOPD and nominates CRP as a potential biomarker for risk-stratifying patients at high risk of adverse short-term outcomes, offering both mechanistic insight and a candidate tool for clinical prognostication.

## 1 Introduction

Chronic Obstructive Pulmonary Disease (COPD) is a highly prevalent and heterogeneous respiratory disorder worldwide, posing a significant public health challenge due to its substantial morbidity and mortality (1, 2). Characterized by persistent airflow limitation, COPD manifests with progressive symptoms such as dyspnea, cough, and sputum production, which severely impair patients’ quality of life (3). According to the Global Burden of Disease Study, COPD was responsible for approximately 3.3 million deaths in 2019, ranking as the third leading cause of death globally (4). With an aging global population, COPD prevalence is projected to rise substantially in the coming decades, potentially leading to over 5.4 million annual deaths from COPD and related conditions by 2060 (5). Acute exacerbations of COPD (AECOPD), often triggered by respiratory infections or environmental exposures, are defined as an acute worsening of respiratory symptoms, including increased dyspnea, sputum volume, and purulence. They represent the most common cause of hospitalization and mortality among COPD patients (6). AECOPD is associated with heightened airway and systemic inflammation, accelerating lung function decline, worsening quality of life, and increasing the risk of death (7). Studies indicate that approximately 46% of COPD patients experience at least one exacerbation per year. AECOPD progresses rapidly, carries a poor prognosis, and is associated with high in-hospital mortality, ranging from 4% to 14% in the Asia-Pacific region, posing a severe threat to patient survival (8).

Notably, Human Immunodeficiency Virus (HIV) infection is an independent risk factor for COPD development (9). Poorly controlled HIV infection, characterized by high viral load or low CD4 cell count, is independently associated with accelerated lung function decline, thereby increasing susceptibility to COPD in this population (10–12). The estimated prevalence of COPD among people living with HIV (PLWH) ranges from 5% to 30%, contributing significantly to reduced quality of life and excess mortality (13, 14). Compared to HIV-uninfected individuals with COPD, PLWH experience more frequent exacerbations, often with greater symptom severity and more pronounced lung function impairment, leading to worse clinical outcomes (15, 16). The co-occurrence of HIV and AECOPD presents a critical yet poorly understood clinical dilemma. The prevailing hypothesis suggests that systemic immune dysregulation caused by HIV infection synergizes with the intense local pulmonary inflammation during AECOPD, fostering a unique pathological state. This synergy likely involves complex interactions among aberrant innate immune signaling, dysregulated adaptive immunity, and alterations in metabolic and oxidative stress pathways. Despite its clinical significance, the precise molecular and immunological landscape defining this high-risk “HIV-AECOPD” phenotype remains largely unexplored (17, 18). While prior studies have implicated certain markers—such as monocyte activation markers sCD163 and TNF-α in diffuse lung injury, and IL-6 and D-dimer in more severe airflow obstruction (19)—there remains a critical lack of specific biomarkers capable of predicting prognosis in this vulnerable population, representing a major gap in current clinical management (20).

Recent advances in functional proteomics and metabolomics have shown promise in identifying novel biomarkers, disease severity indicators, and therapeutic candidates (21). For instance, proteomic markers such as sRAGE, ICAM1, and CCL20 in blood have been associated with lung function, while metabolomic signatures involving purines, sphingomyelins, and glycerophospholipids have been linked to exacerbations (22, 23). However, most prior multi-omics studies have focused on delineating AECOPD subgroups in general, leaving the high-risk population of HIV-coinfected individuals largely overlooked.

To address this knowledge gap, the present study employed an integrated multi-omics strategy with the following objectives: (1) to delineate the specific proteomic and metabolomic profiles associated with HIV-AECOPD; (2) to identify and validate a panel of key differentiating molecules; and (3) to evaluate their clinical utility as biomarkers for prognostic risk prediction. Our findings aim not only to provide novel mechanistic insights into the immunopathology of this complex comorbidity but also to offer a practical, biomarker-driven framework for improving personalized management and outcomes in this high-risk patient population.

## 2 Materials and methods

### 2.1 Study subjects and sample collection

This study consecutively enrolled patients hospitalized for Acute Exacerbation of Chronic Obstructive Pulmonary Disease (AECOPD) in the Department of Respiratory and Critical Care Medicine from January 2024 to December 2025. Based on HIV infection status, patients were divided into two groups: the HIV-associated AECOPD group (HIV-AECOPD group, n=25) and the AECOPD group (n=25). Age- and sex-frequency-matched healthy volunteers were recruited as the healthy control group (Ctrl group, n=25). The inclusion criteria were as follows: (1) HIV-AECOPD group: Aged 40–80 years, clinically diagnosed with AECOPD according to the Global Initiative for Chronic Obstructive Lung Disease (GOLD) criteria, defined as an acute worsening of respiratory symptoms (increased dyspnea, cough, sputum volume, and/or purulence). Confirmed HIV infection by Western blot. No prior systemic corticosteroids or antibiotics for the current exacerbation before admission. (2) AECOPD group: Aged 40–80 years, clinically diagnosed with AECOPD using the same criteria as above. Negative HIV antibody screening and confirmatory test results at admission. (3) Ctrl group: Aged 40–80 years, frequency-matched to the patient groups. No history of chronic respiratory diseases, with normal pulmonary function tests. Negative HIV antibody screening result. No recent history of acute infection, trauma, or surgery. Within each group of 25 subjects, 5 were randomly selected to form the discovery set for multi-omics sequencing analysis, while the remaining 20 subjects in each group served as the independent validation set. Fasting peripheral venous blood was collected from all AECOPD patients within 24 hours of admission, prior to the initiation of any systemic therapy. Fasting blood was collected from healthy controls during morning health examinations. Blood samples were drawn into vacuum tubes containing a clotting activator. Serum was separated by centrifugation at 3000 rpm for 15 minutes at 4 °C within 2 hours of collection. The serum was then aliquoted and immediately stored at -80 °C, with care taken to avoid repeated freeze-thaw cycles, until subsequent omics analysis and biomarker assays.

### 2.2 Proteomic analysis

The serum proteomic analysis was conducted using a data-independent acquisition (DIA) mass spectrometry strategy. In brief, serum proteins were first processed using the filter-aided sample preparation (FASP) method. Total proteins were extracted with sodium deoxycholate (SDC) lysis buffer, followed by reduction with Tris(2-carboxyethyl)phosphine (TCEP) and alkylation with chloroacetamide (CAA). Subsequently, the proteins were digested overnight with trypsin at 37 °C. The resulting peptide mixture was desalted using C18 Stage-Tips prior to LC-MS/MS analysis. MS data acquisition was performed on a platform coupling nanoflow liquid chromatography with a high-resolution mass spectrometer. Peptides were separated on a C18 reverse-phase analytical column using an acetonitrile gradient. For DIA acquisition, the full MS scan resolution was set to 120, 000, the MS/MS resolution to 30, 000, and the scan range was set from 400 to 1100 m/z. The raw MS data were processed and analyzed using DIA-NN software. The search was conducted against the UniProt human proteome database. Key search parameters included: carbamidomethylation of cysteine as a fixed modification; oxidation of methionine and N-terminal acetylation as variable modifications; a maximum of two missed cleavages were allowed. The mass tolerance for both precursor and fragment ions was set to 20 ppm. Protein identification and quantification results were subsequently obtained.

### 2.3 Metabolomic analysis

Untargeted metabolomic profiling of serum samples was performed using an ultra-high-performance liquid chromatography coupled with quadrupole time-of-flight mass spectrometry (UHPLC-QTOF-MS) platform. Briefly, an aliquot of each serum sample was mixed with a methanol-acetonitrile solvent for protein precipitation. The mixture was centrifuged, and the supernatant was collected, vacuum-dried, and then reconstituted in a methanol-water solution. After another centrifugation step, the supernatant was injected for analysis. Chromatographic separation was achieved on an ACQUITY Premier HSS T3 column maintained at 45 °C, using a gradient elution with methanol containing 0.1% formic acid. The total analysis time was 16 minutes. Mass spectrometric data were acquired in data-dependent acquisition (DDA) mode, with alternating scans in both positive and negative ionization modes. The resolution was set to 70, 000 for full MS scans and 17, 500 for MS/MS scans, with a scan range of m/z 150–1500. Raw data were processed using PQI software, which included peak picking, alignment, and baseline correction. Metabolite identification was accomplished by matching against both a proprietary in-house standard library and the public HMDB database. The mass tolerance was set to 12 ppm for precursor ions and 20 ppm for fragment ions. Finally, qualitative identification and relative quantification results for the metabolites were obtained.

### 2.4 Omics data analysis

The quantified protein and metabolite data were normalized. Differentially expressed proteins and metabolites between groups were identified using R software (version 4.5.0) with the following criteria: an adjusted *P*-value < 0.05 and a fold change > 1.2 or < 0.83. Functional enrichment analysis, including KEGG pathway and Gene Ontology (GO) analyses of the differential molecules, was subsequently performed using the DAVID bioinformatics platform.

### 2.5 Protein–metabolite network correlation

To investigate the interaction between the proteome and metabolome, an integrated correlation network was constructed. The quantitative data of all identified differentially expressed proteins and metabolites were integrated. Spearman rank correlation coefficients and their corresponding *P*-value were calculated for each protein–metabolite pair using R software (version 4.5.0). To control for the false positive rate resulting from multiple hypothesis testing, all correlation *P*-value were adjusted using the false discovery rate (FDR) method to generate FDR values. Protein–metabolite pairs with |Spearman’s ρ| > 0.8 and FDR < 0.05 were defined as significantly correlated and retained for subsequent network construction. Based on the filtered significant correlation pairs, a protein–metabolite interaction network was visualized using Cytoscape software (version 3.7.2). In the network visualization: nodes represent proteins or metabolites, and both node size and color depth are mapped according to their degree, with higher-degree nodes appearing larger and darker in color. Edges represent significant correlations between proteins and metabolites. Edge width is proportional to the absolute value of the Spearman correlation coefficient, visually indicating correlation strength. Edge color is gradient-shaded according to the FDR value, with darker colors corresponding to smaller FDR values.

### 2.6 Biomarker validation experiments

Based on the results of multi-omics analysis and inter-omics correlation networks, key candidate molecules were selected for quantitative measurement in peripheral blood samples from different groups. In an independent, expanded validation cohort (n=20 per group), serum concentrations of amyloid A (SAA) and C-reactive protein (CRP) were measured using an automated clinical chemistry analyzer. The levels of total triglycerides (TG), immunoglobulin G (IgG), complement C3, and complement C4 were quantified using a biochemical analyzer. Serum levels of tumor necrosis factor-alpha (TNF-α) and interleukin-6 (IL-6) were measured using enzyme-linked immunosorbent assay kits. All experimental procedures were performed strictly according to the manufacturers’ instructions.

### 2.7 Clinical data analysis

Patients were followed up for 3 months via telephone or outpatient visit after discharge. They were categorized into a poor prognosis group or a good prognosis group. Poor prognosis was defined as disease recurrence, re-hospitalization due to respiratory causes, occurrence of severe complications (such as cor pulmonale or respiratory failure), or death. Patients not meeting these criteria were classified as having a good prognosis.

### 2.8 Statistical analysis

Statistical analyses were performed using SPSS software (version 26.0) and R software (version 4.5). The normality of continuous variables was first assessed using the Shapiro-Wilk test. Variables conforming to a normal distribution are presented as mean ± standard deviation (SD); non−normally distributed continuous variables are presented as median (Q1, Q3). For normally distributed continuous variables, comparisons among multiple groups were conducted using one-way analysis of variance (ANOVA) followed by Tukey’s honest significant difference (HSD) *post-hoc* test, and the Student’s t-test was used for comparisons between two groups. For non-normally distributed continuous variables, the Kruskal-Wallis test was used for multi-group comparisons, and the Wilcoxon rank-sum test was used for two-group comparisons. In the validation assays involving multiple biomarkers, we applied Bonferroni correction to adjust *P*-value for multiple comparisons, with an adjusted *P*-value < 0.05 considered statistically significant. Categorical variables were compared using the chi-square test. The Spearman’s rank correlation analysis was employed to assess the correlations between biomarker levels and disease severity grades. A random forest model was applied to evaluate the importance of each biomarker in predicting prognosis. Receiver operating characteristic (ROC) curves were plotted to evaluate the predictive efficacy of the biomarkers for poor prognosis, and the area under the curve (AUC) was calculated. A *P*-value of less than 0.05 was considered statistically significant.

## 3 Result

### 3.1 Clinical characteristics of the study population

**Figure 1:**
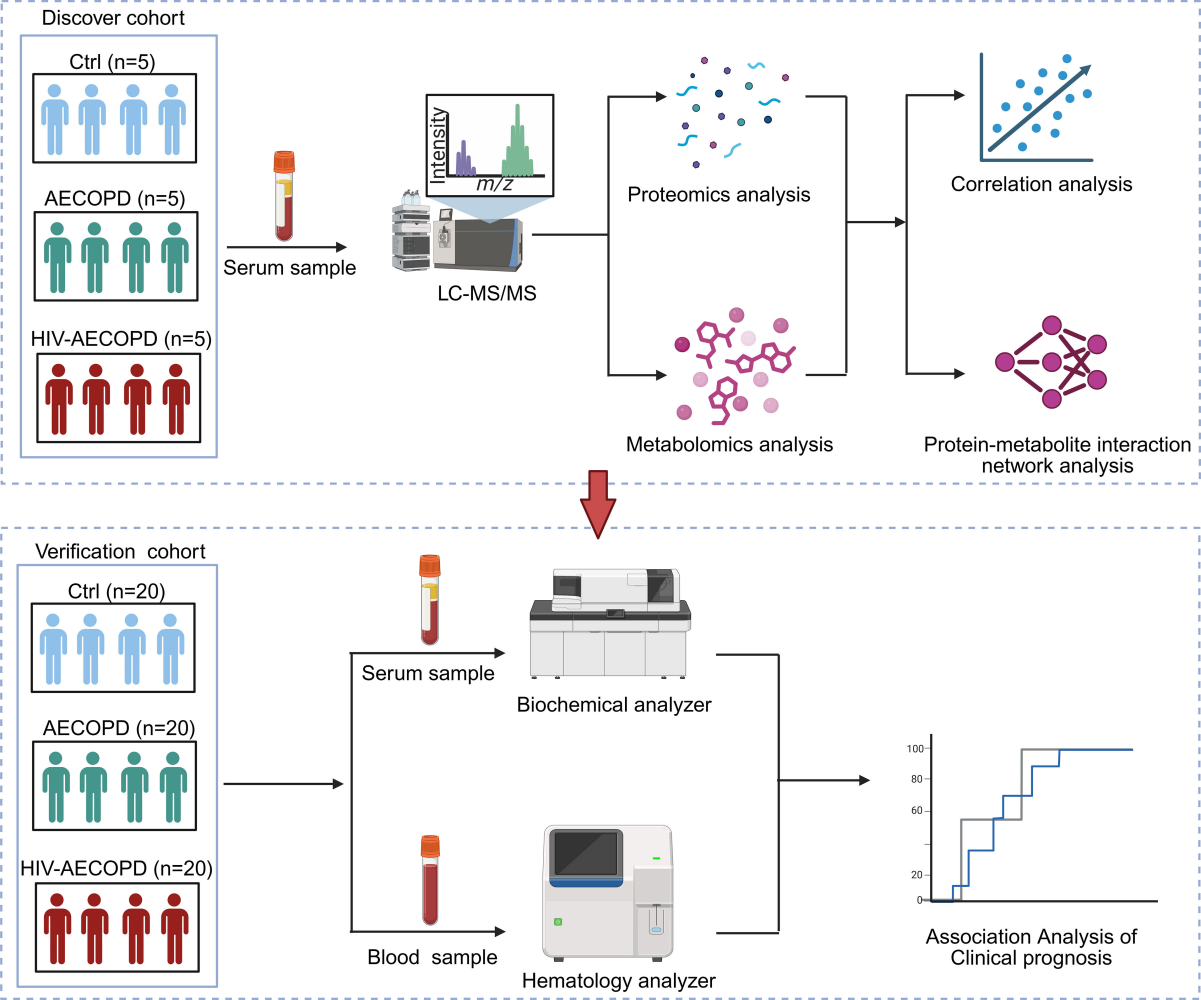
Graphical overview of the study workflow.

A total of seventy-five participants were enrolled in this study, comprising twenty-five patients in the HIV-AECOPD group, twenty-five in the AECOPD group, and twenty-five in the healthy control group. The demographic and baseline clinical characteristics of the three groups are summarized in **Table 1**. As shown, no statistically significant differences were observed among the HIV-AECOPD, AECOPD, and control groups regarding age, sex distribution, or body mass index (BMI) (all *P* > 0.05), indicating well-balanced comparability for these fundamental characteristics. Both the HIV-AECOPD and AECOPD groups had significantly higher proportions of current/former smokers and greater smoking pack-years compared with the healthy control group (both *P* < 0.001). For COPD-related clinical indices, patients in the HIV-AECOPD group had a significantly higher number of acute exacerbations in the year prior to enrollment compared to those in the AECOPD group (2.60 ± 1.08 vs. 2.04 ± 1.10, P = 0.0039), suggesting that HIV infection may be associated with more frequent acute disease episodes. No significant differences were found in the prevalence of comorbidities, including cardiovascular disease and type 2 diabetes, among the three groups (all *P* > 0.05). Additional clinical characteristics at admission and HIV-related parameters are detailed in **Table 1**. The research workflow is shown in **Figure 1**.

**Table 1:** Comparison of clinical data of the subjects.

Characteristics	HIV-AECOPD group (*n* = 25)	AECOPD group (*n* = 25)	Healthy control group (*n* = 25)	P-value
Demographics				
Age, years	65.20 ± 7.04	66.28 ± 7.51	64.40 ± 7.84	0.6727
Male, *n* (%)	68% (17/25)	60.00% (15/25)	60.00% (15/25)	0.796
BMI (kg/m²)	24.72 ± 3.17	25.11 ± 3.27	26.08 ± 3.78	0.3533
Current or ever smoker, n (%)	84.00% (21/25)	80.00% (20/25)	36.00% (9/25)	<0.001
Smoking amount, pack-year	34 (17.50, 61.00)	33 (18.50, 57.00)	0(0, 22.50)	<0.001
Admission and hospitalization data				
Body temperature, °C	36.59 ± 0.64	36.65 ± 0.72	NA	0.8617^1^
Lung infection, n (%)	100%(25/25)	100%(25/25)	NA	
Length of hospital stay, days	10.28 ± 3.32	9.76 ± 2.93	NA	0.7897^1^
Exacerbations in previous year	2.60 ± 1.08	2.04± 1.10	NA	0.0039^1^
Comorbidities				
Cardiovascular disease, *n* (%)	36.00% (9/25)	32.00% (8/25)	24.00% (6/25)	0.645
Type 2 Diabetes, *n* (%)	16.00% (4/25)	20.00% (5/25)	12.00% (3/25)	0.743
HIV-related				
HIV viral load (copies/mL)	7485 (4516, 21800)^2^			
CD4+ T cell count (cells/μL)	103.42 ± 73.37			
CD4/CD8 ratio	0.24 ± 0.21			
On ART, %	100			

^1^ Comparison was made between the HIV-AECOPD group and the AECOPD group only, using an independent samples t-test. ^2^Those whose viral load could be detected (n = 6).

### 3.2 Serum proteomic profiling reveals distinct immune and metabolic features in HIV-associated AECOPD

**Figure 2:**
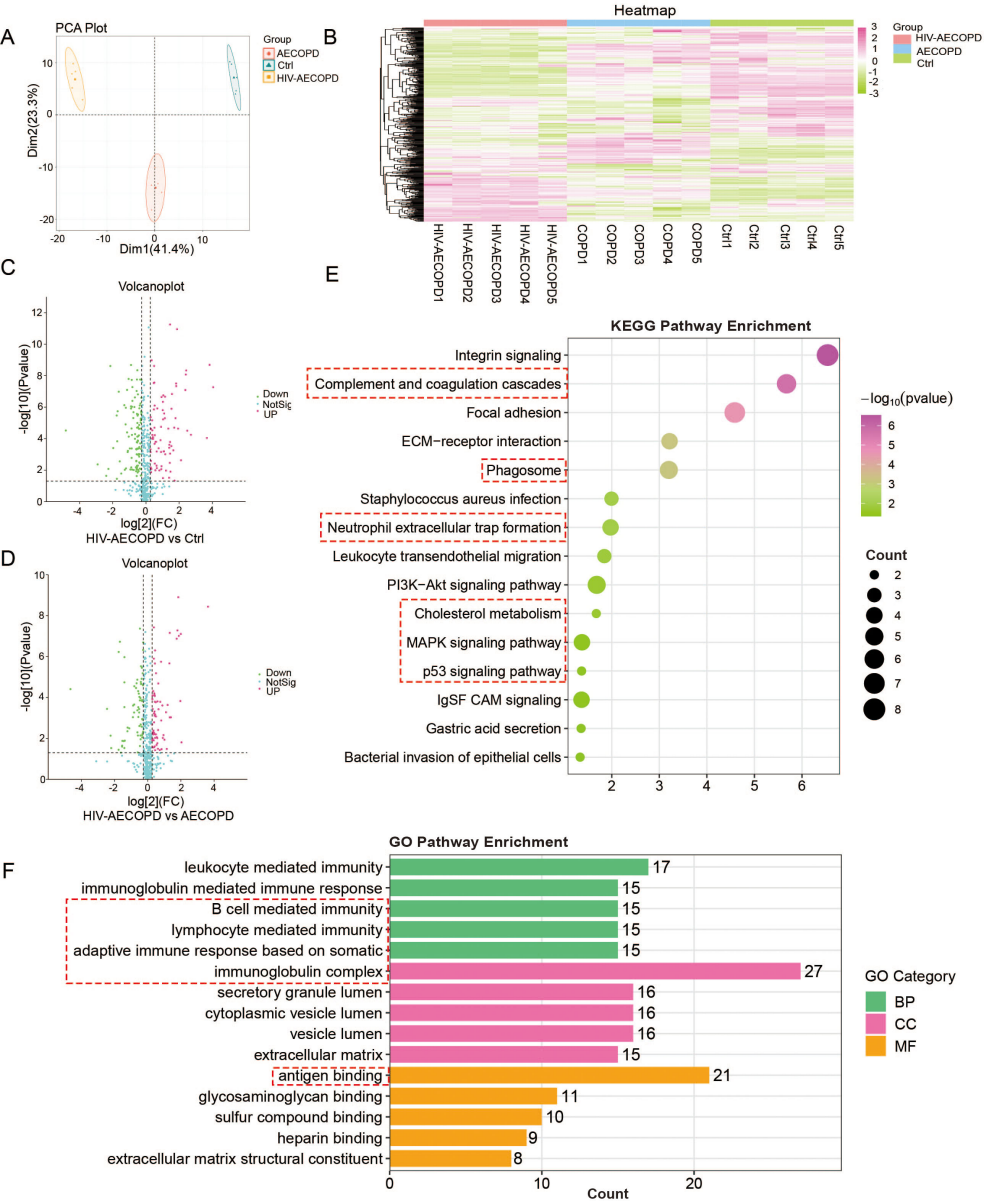
Serum proteomic profiling identifies distinct immune and metabolic signatures in HIV-associated AECOPD. **(A)** Principal component analysis (PCA) plot of serum samples from the HIV-AECOPD, AECOPD, and healthy control (Ctrl) groups, showing distinct clustering. **(B)** Heatmap depicting the expression patterns of all identified proteins across the three groups. **(C, D)** Volcano plots displaying differentially expressed proteins (DEPs) in the comparison of the HIV-AECOPD group versus the Ctrl group **(C)** and versus the AECOPD group **(D)**. Red and green dots represent significantly upregulated and downregulated proteins. **(E)** KEGG pathway enrichment analysis of common DEPs shared between the two comparisons (HIV-AECOPD vs. Ctrl and HIV-AECOPD vs. AECOPD). The top 15 enriched pathways are shown. Key pathways related to immune dysregulation and metabolic alteration are highlighted in red. **(F)** Gene Ontology (GO) enrichment analysis of the common DEPs. Top terms within each category are displayed.

To delineate the unique serum proteomic characteristics of patients with HIV-associated AECOPD, we first performed an unbiased proteomic analysis of samples from the HIV-AECOPD, AECOPD, and healthy control (Ctrl) groups. Principal component analysis (**Figure 2A**) showed clear separation among the three groups, indicating substantial differences in their overall protein expression profiles. Notably, a discernible distance was also observed between the HIV-AECOPD and AECOPD groups, suggesting that HIV infection introduces additional and distinguishable alterations to the AECOPD proteomic landscape. A heatmap constructed from the expression levels of all identified proteins (**Figure 2B**) further visualized the global expression differences across groups, revealing distinct clusters of proteins that were consistently up- or down-regulated, thereby providing a basis for subsequent differential analysis. To precisely identify the proteins driving these changes, pairwise comparisons were conducted. Compared to the Ctrl group, the HIV-AECOPD group exhibited 81 up-regulated and 111 down-regulated differentially expressed proteins (DEPs) (**Figure 2C**). Similarly, compared to the AECOPD group, the HIV-AECOPD group showed 72 up-regulated and 69 down-regulated DEPs (**Figure 2D**). To focus on the core pathophysiological processes in HIV-AECOPD, we performed pathway enrichment analysis on the DEPs common to both comparisons. KEGG enrichment analysis (**Figure 2E**) revealed the top 15 significantly enriched pathways. Key among these were Complement and coagulation cascades, Phagosome, Neutrophil extracellular trap formation, Cholesterol metabolism, MAPK signaling pathway, and p53 signaling pathway. This pattern implicates crucial roles for innate immune activation, dysregulated lipid metabolism, and cellular stress response in the disease. Gene Ontology (GO) enrichment analysis (**Figure 2F**) characterized these common DEPs across biological processes (BP), cellular components (CC), and molecular functions (MF). The DEPs were strongly enriched in processes such as B cell mediated immunity and lymphocyte mediated immunity, and were localized to the immunoglobulin complex. Collectively, these findings suggest that complement system activation, lipid metabolism disturbance, and abnormal humoral immune responses constitute central pathological features of HIV-AECOPD.

### 3.3 Functional enrichment analysis reveals the overall disease effect and HIV-specific immunological dysregulation in AECOPD

**Figure 3:**
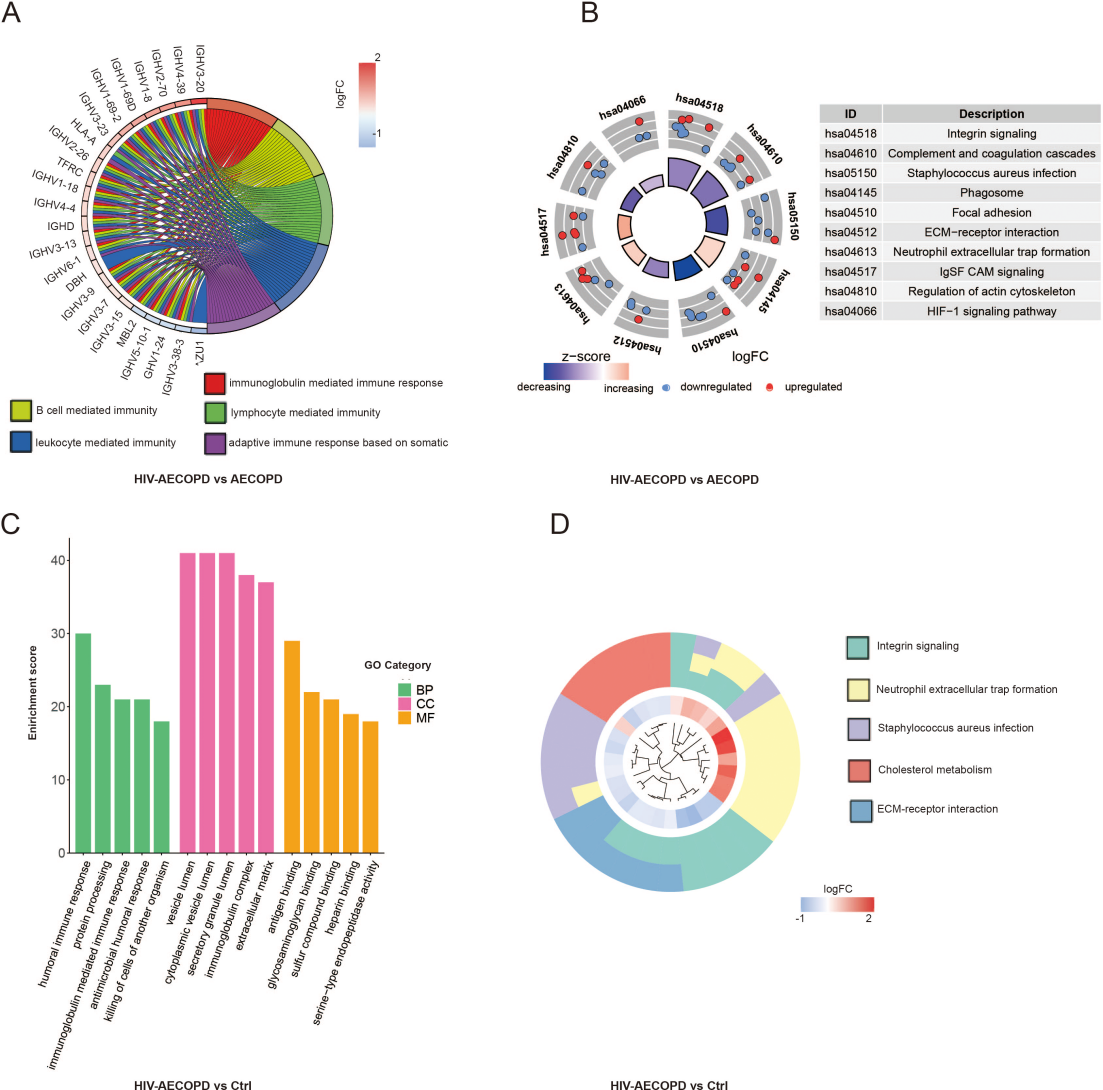
Functional enrichment analysis of differentially expressed proteins in HIV-AECOPD. **(A)** Bar plot of GO enrichment analysis for differentially expressed proteins (DEPs) in the HIV-AECOPD group versus the healthy control (Ctrl) group, showing the top 5 significantly enriched terms per category. **(B)** Circular cluster plot of KEGG pathway enrichment analysis for DEPs from the HIV-AECOPD vs. Ctrl comparison, displaying the top 5 enriched pathways. **(C)** Chord diagram illustrating GO enrichment analysis (BP) for DEPs in the HIV-AECOPD group compared to the AECOPD group, highlighting the top 5 terms. **(D)** Circular cluster plot of KEGG pathway enrichment analysis for DEPs from the HIV-AECOPD vs. AECOPD comparison, showing the top 10 enriched pathways.

To dissect the proteomic features of HIV-AECOPD in greater depth, we performed separate functional enrichment analyses on differentially expressed proteins (DEPs) comparing the HIV-AECOPD group to the healthy control (Ctrl) and to the AECOPD group. This approach aimed to distinguish the “overall disease effect” from the “HIV-specific additive effect.” **Figures 2A, B** focus on the overall alterations in HIV-AECOPD compared to the healthy state. GO analysis (**Figure 3A**) revealed that the DEPs were significantly enriched in processes such as the killing of cells of other organisms, immunoglobulin-mediated immune response, and antimicrobial humoral response. These proteins were localized to the extracellular matrix and immunoglobulin complex, with molecular functions dominated by serine-type endopeptidase activity and antigen binding. These findings indicate that, relative to health, patients with HIV-AECOPD are in a state of intense systemic immune activation, characterized by predominant humoral immunity and antimicrobial defense, and accompanied by potential alterations in the tissue microenvironment. KEGG pathway analysis (**Figure 3B**) corroborated and extended this discovery. The enriched pathways not only included immune-inflammatory pathways such as integrin signaling, neutrophil extracellular trap formation, and *Staphylococcus aureus* infection, but also revealed the cholesterol metabolism pathway, suggesting that metabolic reprogramming is closely associated with this disease state. **Figures 3C, D** elucidate the unique contribution of HIV infection in the context of AECOPD. The GO analysis results (**Figure 3**) were particularly striking, with the top five enriched terms exclusively concentrated on B cell mediated immunity, lymphocyte mediated immunity, and adaptive immune response based on somatic recombination. This strongly suggests that aberrantly activated adaptive humoral immunity is the most distinctive feature separating HIV-AECOPD from AECOPD alone. KEGG analysis (**Figure 3D**) not only recapitulated key innate immunity and inflammation pathways like the complement and coagulation cascades, phagosome, and NET formation, but also enriched two new pathways with significant functional implications: Regulation of actin cytoskeleton and HIF-1 signaling pathway. The former is directly linked to critical immune cell functions such as migration and phagocytosis, while the latter connects hypoxia, inflammation, and cellular metabolism, providing crucial insights into how HIV may remodel immune cell function and metabolic status.

### 3.4 Metabolomic analysis reveals extensive metabolic reprogramming in patients with HIV-AECOPD

**Figure 4:**
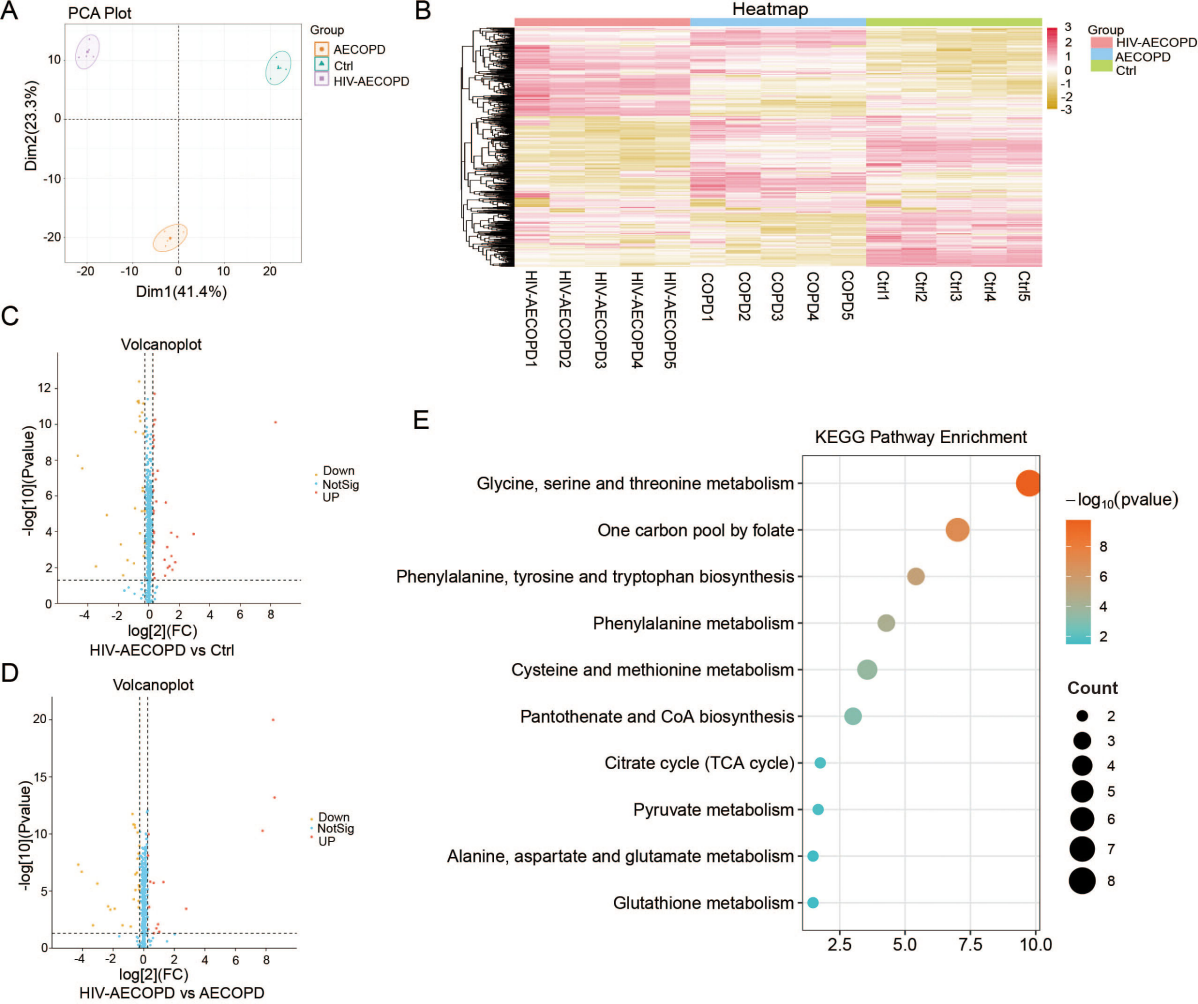
Metabolomic profiling reveals extensive metabolic reprogramming in HIV-AECOPD patients. **(A)** Principal component analysis (PCA) plot of serum metabolomic profiles from the HIV-AECOPD, AECOPD, and healthy control (Ctrl) groups, showing distinct separation among groups. **(B)** Heatmap depicting the relative abundance of all identified metabolites across the three study groups. **(C, D)** Volcano plots of differentially expressed metabolites (DEMs) in comparisons of the HIV-AECOPD group versus the Ctrl group **(C)** and versus the AECOPD group **(D)**. Significantly upregulated and downregulated metabolites are highlighted. **(E)** Bubble plot of the top 10 KEGG pathways enriched by the common DEMs shared between the two comparisons (HIV-AECOPD vs. Ctrl and HIV-AECOPD vs. AECOPD).

To systematically elucidate the characteristics of metabolic reprogramming in patients with HIV-AECOPD, we performed untargeted metabolomic profiling of serum samples from all three groups. Principal component analysis (PCA) (**Figure 4A**) demonstrated a clear separation trend in the metabolomic profiles among the HIV-AECOPD, AECOPD, and Ctrl groups, indicating significant differences in their overall metabolic states. This finding corroborated the results from the proteomic analysis. The global metabolite expression heatmap (**Figure 4B**) further visualized a large number of metabolites that exhibited consistent changes across groups, forming the basis of a disease-specific metabolic fingerprint. To identify key metabolic perturbations, pairwise comparisons were conducted. Compared to the Ctrl group, the HIV-AECOPD group exhibited 32 up-regulated and 27 down-regulated differentially expressed metabolites (DEMs) (**Figure 4C**). Similarly, compared to the AECOPD group, the HIV-AECOPD group showed 15 up-regulated and 26 down-regulated DEMs (**Figure 4D**). To focus on the most central metabolic alterations in HIV-AECOPD, we performed pathway enrichment analysis on the DEMs common to both comparisons. KEGG analysis (**Figure 4E**) showed that these core DEMs were significantly enriched in 10 key metabolic pathways, which could be categorized into three main functional modules: Amino Acid and One-Carbon Metabolism: This included Glycine, serine and threonine metabolism; One carbon pool by folate; Phenylalanine, tyrosine and tryptophan biosynthesis; and Cysteine and methionine metabolism. These pathways provide essential nucleotide precursors and methyl donors to support the rapid proliferation and functional activity of immune cells. Energy and Central Carbon Metabolism: Pathways such as the Citrate cycle (TCA cycle), Pyruvate metabolism, and Alanine, aspartate and glutamate metabolism were enriched, indicating a reprogramming of cellular energy metabolism and biosynthetic demands. Antioxidant Defense: The significant enrichment of the Glutathione metabolism pathway was particularly critical, directly reflecting the activation of a core antioxidant system in response to oxidative stress.

### 3.5 Integrated multi-omics correlation network identifies core hub proteins and metabolites in HIV-AECOPD

**Figure 5:**
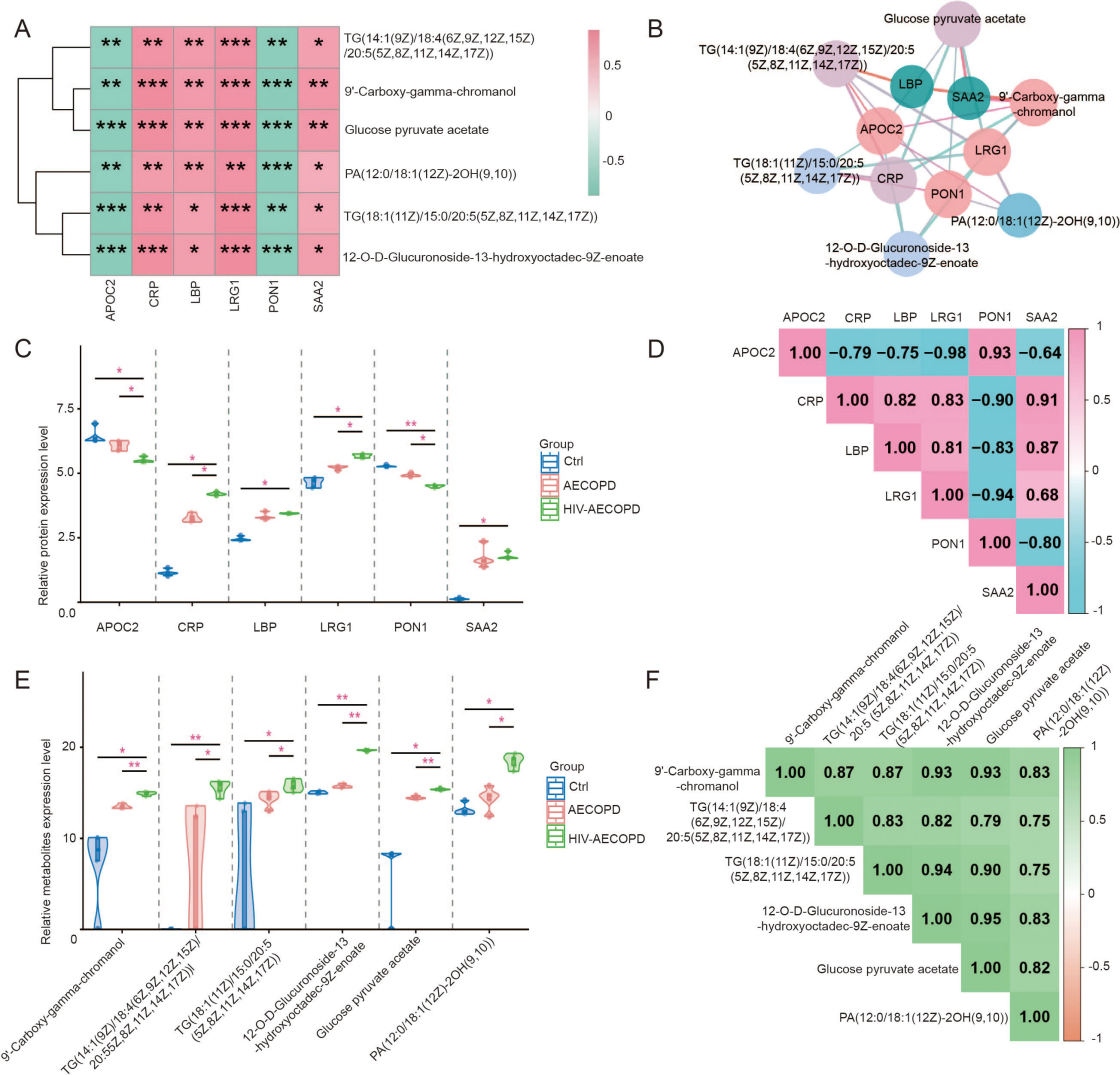
Integrated multi-omics correlation network identifies core hub proteins and metabolites in HIV-AECOPD. **(A)** Heatmap of Spearman correlation coefficients between key proteins and metabolites identified from integrated analysis. Color intensity represents the strength and direction of correlation. **(B)** Protein–metabolite correlation network visualization of the six key proteins and their paired metabolites, constructed based on the strongest correlations identified in **(A)**. Edge thickness corresponds to correlation strength. The color of the edge corresponds to its significance. Red indicates high significance, while blue indicates low significance. **(C)** Relative expression profiles of the six core proteins (APOC2, CRP, LBP, LRG1, PON1, SAA2) across the HIV-AECOPD, AECOPD, and Ctrl groups. **(D)** Correlation matrix (Spearman) among the six core proteins themselves, revealing co-expression patterns within the protein module. **(E)** Relative abundance profiles of the six paired metabolites across the three study groups. **(F)** Correlation matrix (Spearman) among the six core metabolites, demonstrating co-variation patterns within the metabolite module. Statistical significance: *P < 0.05, **P 0.01, ***P 0.001.

To extract the most critical molecules driving the HIV-AECOPD phenotype from the complex omics data, we performed a cross-omics Spearman correlation network analysis between the differentially expressed proteins and metabolites to identify hub molecule pairs that co-vary. We calculated correlations between DEPs and DEMs and visualized the pairs with the strongest correlations (|Spearman’s ρ| > 0.8). The correlation heatmap (**Figure 5A**) clearly delineated a tightly connected network comprising six core proteins and six core metabolites. Notably, these correlations displayed a highly coordinated and directionally consistent pattern: pro-inflammatory proteins (CRP, LBP, LRG1, SAA2) showed strong positive correlations with all metabolites, whereas the antioxidant protein PON1 and the apolipoprotein APOC2 exhibited strong negative correlations with all metabolites. Based on these strongest correlations, a protein-metabolite interaction network was constructed (**Figure 5B**). The core of this network primarily consisted of key hub molecules representing the features of ‘inflammatory storm’, ‘lipid dysregulation’, and ‘complement activation’, specifically including the acute inflammation markers CRP and SAA, the lipid transporter APOC2 and specific triglycerides (TG), as well as LBP and LRG1 which are associated with complement and bacterial responses. The expression profiles of these hub molecules further confirmed their disease specificity. **Figure 5C** shows that the levels of pro-inflammatory proteins (CRP, SAA2, LBP, LRG1) were highest in the HIV-AECOPD group, followed by the AECOPD group, and lowest in the Ctrl group. In contrast, PON1and APOC2 showed an inverse trend, with its lowest expression in the HIV-AECOPD group. **Figure 4E** indicates that the relative abundance of all six key metabolites was significantly higher in the HIV-AECOPD group compared to the other two groups. Finally, we analyzed the co-variation patterns within each molecular module. The inter-protein correlation matrix (**Figure 5D**) revealed strong positive correlations among the pro-inflammatory proteins themselves and strong negative correlations with PON1 and APOC2, forming a highly coordinated protein co-expression module. Similarly, the inter-metabolite correlation matrix (**Figure 5F**) demonstrated extensive and strong positive correlations among the metabolites, constituting a tightly coupled metabolite co-variation module.

### 3.6 Validation of key biomarkers in an independent cohort and their association with clinical prognosis

**Figure 6:**
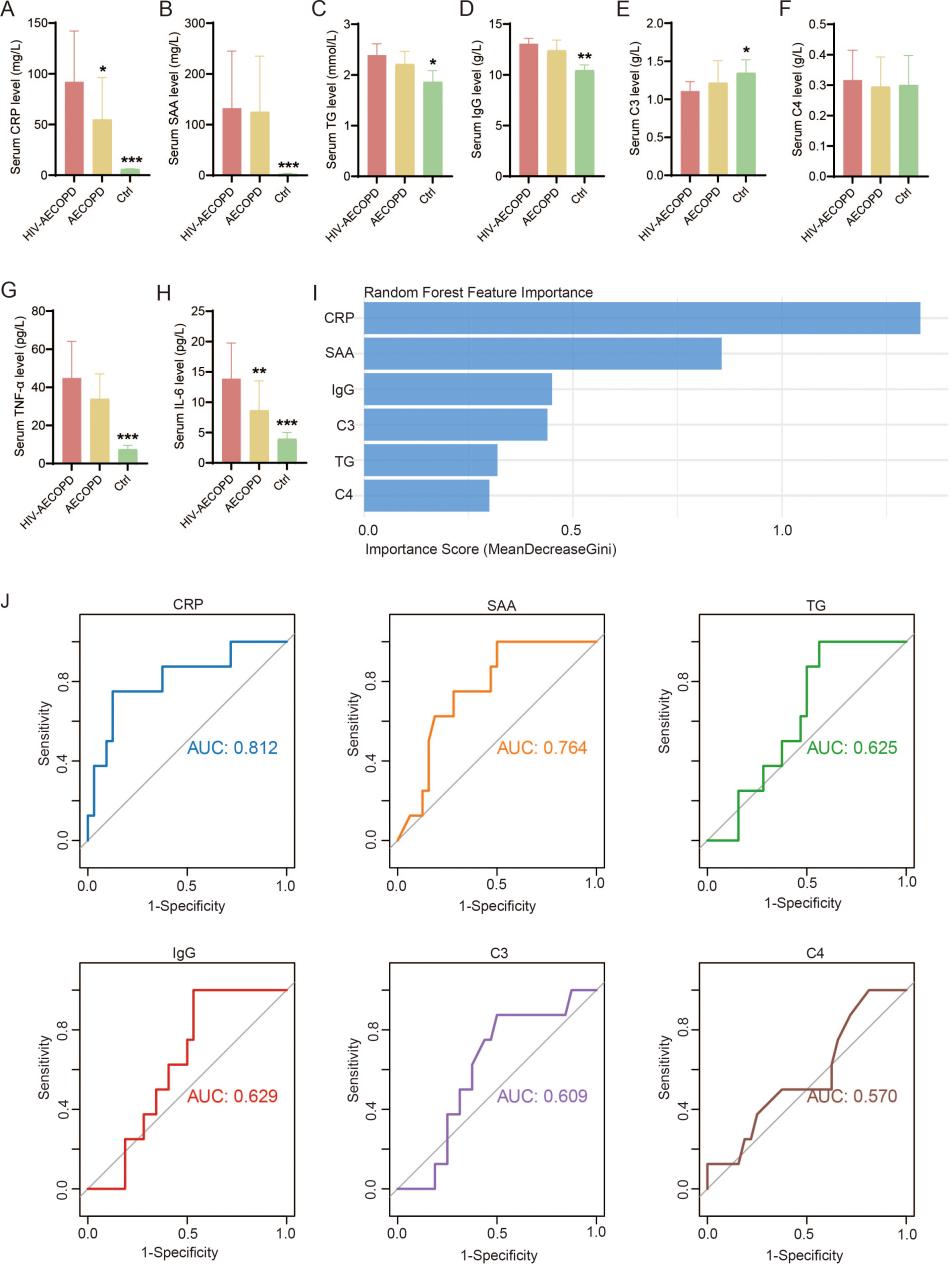
Validation of candidate biomarkers in an independent cohort and assessment of their prognostic value. **(A–F)** Bar graphs showing the distribution of serum levels for CRP, SAA, TG, IgG, C3, and C4 across the HIV-AECOPD (n=20), AECOPD (n=20), and healthy control (Ctrl, n=20) groups in the independent validation cohort. Statistical significance among groups was determined by one-way ANOVA (*P < 0.05, **P < 0.01, ***P < 0.001). **(G, H)** Serum levels of TNF-α and IL-6 in the three groups of the independent validation cohort. **(I)** Variable importance plot derived from a Random Forest model constructed to predict 3-month poor prognosis. The importance of each biomarker is quantified by the Mean Decrease in Gini index. **(J)** Receiver operating characteristic (ROC) curves evaluating the predictive performance of each biomarker for poor 3-month prognosis. The area under the curve (AUC) is displayed for each marker. Statistical significance: *P < 0.05, **P < 0.01, ***P < 0.001.

To validate the core pathological features suggested by the multi-omics analyses, we quantitatively measured the six candidate biomarkers (CRP, SAA, TG, IgG, C3, C4) that were selected based on the aforementioned mechanisms in an independent, expanded validation cohort (n=20 per group). The validation results (**Figures 6A–F**) successfully recapitulated the trends indicated by the omics analysis and delineated a clear spectrum of disease-associated changes. Serum levels of the acute-phase proteins SAA and CRP exhibited a significant gradient across the three groups: HIV-AECOPD > AECOPD > healthy controls. Levels of immunoglobulin G (IgG) and total triglycerides (TG) also showed an increasing trend in the HIV-AECOPD group. In stark contrast, the levels of the complement components C3 displayed an inverse gradient: HIV-AECOPD < AECOPD < healthy controls. The level of complement component C4 showed no significant difference among all groups. In addition to these six core biomarkers, we measured two classical pro-inflammatory cytokines, TNF-α and IL-6, in the same validation cohort (**Figures 6G, H**). The levels of both cytokines were significantly elevated in the HIV-AECOPD group compared with the control group. Moreover, the increase in IL-6 was more pronounced in the HIV-AECOPD group than in the AECOPD-alone group. Next, we evaluated the predictive value of these biomarkers for the prognosis of AECOPD patients in the validation cohort. Analysis using a Random Forest model (**Figure 6I**) revealed that for predicting poor 3-month prognosis, CRP had the highest predictive importance (Mean Decrease Gini = 1.33), followed by SAA, reaffirming the central role of these two inflammatory markers. ROC curve analysis (**Figure 6J**) further quantified their predictive performance. CRP demonstrated a good predictive ability (AUC = 0.812, 95% CI: 0.628–0.997), and SAA also showed considerable predictive value (AUC = 0.764, 95% CI: 0.604–0.923). The predictive value of the other biomarkers (TG, IgG, C3, C4) was relatively limited (AUC ranging from 0.57 to 0.63).

## 4 Discussion

In this study, we employed an integrated approach combining proteomics, metabolomics, and systematic clinical validation to delineate, for the first time, the distinct landscape of immune–metabolic dysregulation in the serum of patients with HIV-associated AECOPD, and successfully identified circulating biomarkers with significant prognostic value. Our proteomic data consistently and strongly point to aberrant activation of B-cell and humoral immunity. This finding aligns with previous studies demonstrating that HIV infection leads to chronic immune activation, B−cell dysfunction, and hypergammaglobulinemia (24–26). In the context of AECOPD, this chronic immune dysregulation may be further amplified. Persistent antigenic stimulation—driven by bacterial infection or viral replication—likely promotes polyclonal B−cell activation and elevated immunoglobulin (e.g., IgG) levels, leading to the formation of abundant immune complexes (27). In patients with acute exacerbation of COPD, complement activation was primarily manifested as significant consumption of C3, whereas C4 levels did not show comparable changes, consistent with earlier reports (28).

Accumulating evidence has established that oxidative stress is intimately involved in the pathogenesis of both HIV and COPD (29–31). Increased oxidative stress frequently contributes to lung function decline in COPD, and the presence of HIV further exacerbates this burden. Our metabolomic analysis revealed extensive metabolic reprogramming, highlighted by significant enrichment of glutathione metabolism and downregulation of the antioxidant enzyme PON1, collectively indicating a severe state of oxidative stress. Furthermore, upregulation of amino acid and one−carbon metabolism may provide essential biosynthetic precursors to support the proliferation of highly activated immune cells. Alterations in cholesterol metabolism suggest an intertwining of lipid dysregulation with inflammatory processes. These metabolic changes are tightly coupled with the inflammatory and immune activation signals uncovered by proteomics, collectively constituting an integrated “immune–metabolic” interaction network.

Notably, by integrating proteomic and metabolomic data through correlation network analysis, we identified a highly coordinated “immune–metabolic co−dysregulation” network specific to HIV−AECOPD, providing direct evidence for the coupling of metabolic disturbances and inflammatory signals. We observed that acute−phase proteins (CRP, SAA2, LBP, LRG1) were significantly positively correlated with oxidized lipid derivatives. These proteins are established markers of systemic inflammation and innate immune activation, and are upregulated in various inflammatory conditions (32–35). Their expression was markedly elevated under the dual insult of persistent HIV−induced immune activation and acute exacerbation of COPD, a finding corroborated by our validation cohort demonstrating significantly increased CRP and SAA levels in HIV−AECOPD patients. Extending this inflammatory profiling, we also measured the pro-inflammatory cytokines TNF-α and IL-6. Both cytokines were significantly elevated in the HIV-AECOPD group compared with healthy controls. Moreover, IL-6 levels were markedly higher in the HIV-AECOPD group than in the AECOPD-alone group, whereas TNF-α showed no significant difference between the two patient groups. The formation of acute-phase reactants is strongly induced by cytokines such as IL-6, and CRP levels often rise in parallel with IL-6 during infection, making IL-6 a potential serum marker for assessing AECOPD severity (36, 37). Furthermore, IL-6 can affect lipid metabolism and promote an increase in triglyceride (TG) levels (38). HIV infection itself leads to chronic elevation of IL-6, and when the acute inflammation of AECOPD is superimposed, an IL-6-driven vicious cycle linking inflammation and lipid metabolism is established (39). Accumulation of triglycerides (TG) has been implicated as a driver of both pulmonary and systemic inflammation (40); the elevated TG levels observed in our validation cohort are consistent with the well−recognized phenotype of HIV−associated hypertriglyceridemia (41).

Critically, the lipid metabolism regulator APOC2 and the antioxidant protein PON1 also displayed broad and significant correlations with the aforementioned lipid metabolites. APOC2 serves as a key activator of lipoprotein lipase, and its dysfunction impairs TG clearance (42). PON1, which binds to high−density lipoprotein (HDL), exerts antioxidative protection; its activity is compromised in settings of chronic inflammation and oxidative stress (43–45). The correlation patterns identified in our study suggest that, in patients with HIV−AECOPD, chronic inflammation and oxidative stress may cooperatively impair PON1 activity while concurrently disrupting APOC2−mediated lipid clearance, leading to accumulation of pro−inflammatory lipids and compromised antioxidant defense. This imbalance is likely amplified when two chronic inflammatory conditions—HIV and COPD—coexist. Additionally, the strong correlations of the vitamin E metabolite 9′−carboxy−gamma−chromanol with CRP, LRG1, and PON1 further point to concomitant compensatory activation and functional impairment of the antioxidant defense system. The robust associations between the energy metabolite glucose−pyruvate−acetate and all six core proteins underscore extensive crosstalk among glycolysis, inflammation, and lipid metabolism, likely reflecting systemic metabolic remodeling under conditions of immune activation.

At the clinical translational level, our study yielded findings with direct clinical applicability. Using random forest modeling and ROC analysis, we demonstrated that CRP is the most important predictor of 3−month readmission or complications in these patients (AUC = 0.812). This observation aligns with the well−documented value of CRP in general COPD management. CRP, a hepatic acute−phase protein predominantly driven by IL−6, has a relatively long half−life and may more stably reflect cumulative inflammatory burden and tissue damage over time (46). Baseline circulating CRP levels are significantly higher in COPD patients than in non−COPD individuals, underscoring its association with systemic inflammation in COPD (47). In acute exacerbations, elevated admission CRP levels correlate with prolonged hospitalization (48); during stable phases, baseline CRP serves as a prognostic biomarker for future exacerbation frequency (49–51). CRP may also aid in distinguishing bacterial from non−bacterial exacerbations and serve as a predictor of bacterial infection risk (52, 53). These clinical utilities likely stem from the broad associations of CRP with key disease phenotypes: studies have shown that CRP levels are positively correlated with prior−year exacerbation frequency, COPD Assessment Test (CAT) scores, and modified Medical Research Council (mMRC) dyspnea scores, and negatively correlated with FEV_1_% predicted and FEV_1_/FVC ratio (54). Our findings further support CRP as a pivotal biomarker for risk stratification in AECOPD and, importantly, demonstrate that its prognostic performance remains robust—and perhaps even more critical—in the complex immunological milieu of HIV co−infection. These results advocate for the adoption of CRP as a point−of−discharge risk assessment tool to identify high−risk HIV−AECOPD patients warranting intensified follow−up and intervention.

Several limitations of this study should be acknowledged. First, the sample size is relatively small (n=75), which reduces statistical power, increases the risk of false positives in omics analyses. Although we performed independent validation in a separate cohort, the limited sample size remains a concern. Second, the study was conducted at a single center, which may introduce selection bias and limit the generalizability of our findings. Therefore, larger, multi-center prospective cohorts are needed to validate our conclusions. Third, while CRP was identified as a promising prognostic biomarker (AUC = 0.8125), the 95% confidence interval is wide (0.628–0.997), indicating considerable uncertainty in this estimate. This reflects the limited sample size and suggests that the true predictive accuracy of CRP should be interpreted with caution until confirmed in larger cohorts. Fourth, the lack of longitudinal biomarker assessment limits our ability to evaluate dynamic changes in biomarker levels and their relationship to disease trajectory. Finally, the higher frequency of acute exacerbations observed in the HIV-AECOPD group suggests possible underlying pathogen-specific susceptibility or host defense deficits not fully elucidated by our omics profiling, warranting further investigation.

In summary, this study systematically characterizes the immune–metabolic dysregulation—characterized by aberrant humoral immunity, complement consumption, oxidative stress, and metabolic reprogramming—that underpins HIV−AECOPD. Beyond elucidating potential pathophysiological mechanisms, we identified CRP as a readily accessible biomarker for short−term prognostic risk stratification in this high−risk population. These findings provide both mechanistic insights and a practical tool to facilitate personalized management and improve clinical outcomes. Future research should aim to directly validate complement activation mechanisms and explore therapeutic strategies targeting these distinct pathological nodes.

## Data Availability

The proteomics and metabolomics data generated in this study are associated with ongoing unpublished research and are currently being further analyzed. Requests for access to these datasets should be directed to the corresponding author.
